# Diabetes and Risk of Post-Fragility Hip Fracture Outcomes in Elderly Patients

**DOI:** 10.1155/2020/8146196

**Published:** 2020-04-14

**Authors:** Wenqing Tian, Jueli Wu, Tao Tong, Lu Zhang, Aiguo Zhou, Ning Hu, Wei Huang, Bo Zhou

**Affiliations:** ^1^Department of Endocrinology, The First Affiliated Hospital of Chongqing Medical University, Chongqing 400016, China; ^2^Department of Orthopaedics, The First Affiliated Hospital of Chongqing Medical University, Chongqing 400016, China

## Abstract

**Objective:**

To explore the effect of diabetes on short-term (30 days after fracture) and 1-year outcomes for fragility hip fracture patients.

**Methods:**

We conducted a retrospective cohort study involving 161 diabetic hip fracture patients (older than 60 years) and 483 nondiabetic hip fracture patients. Patients were followed up on day 30 and 1 year after fracture. The short-term outcome was complications that occurred within 30 days after hip fracture and length of stay. The 1-year outcomes were postfracture functional outcomes and reduced activity level and mortality rate within 1 year after fracture. The clinical characteristics and outcomes of patients were analyzed.

**Results:**

Compared with nondiabetic patients, diabetic patients had a longer length of awaiting surgery (6.0 vs. 5.0 days, *P*=0.031) and a longer length of total hospital stay (17 vs. 15 days, *P* < 0.001). Furthermore, compared with nondiabetic patients, diabetic patients have higher costs (*P*=0.011), in addition to being more prone to developing urinary tract infections (6.2% vs. 1.7%, *P*=0.002) and deep vein thrombosis (4.3% vs. 1.4%, *P*=0.029) complications. However, at one-year follow-up, no differences in recovery of function and mortality were observed between the two groups.

**Conclusions:**

Diabetic patients are at an increased risk of urinary tract infections and deep vein thrombosis complications but have similar recovery of function and 1-year mortality compared to nondiabetic patients.

## 1. Introduction

Osteoporotic hip fractures are frequent in older people and represent a worldwide challenge [1]. Despite the declining trend in the incidence of hip fracture in western populations, some studies conducted in Asia still showed an increase in the age-adjusted incidence rates of hip fractures [2]. More importantly, compelling studies have demonstrated that most older people who have undergone fragility hip fractures have more comorbidities, poorer functional outcomes, and increased 1-year mortality [3, 4]. Nonetheless, data from Asia remain limited.

Diabetes is a worldwide epidemic [5] that is a common comorbid condition in geriatric adults and may complicate recovery from hip fracture [6–8]. Although the association between both type 1 and type 2 diabetes and increased risk of hip fracture is well documented [9–11], a number of issues regarding the effect of diabetes on complications and functional recovery in patients after hip fractures remain to be clarified. It has been demonstrated that the activities of daily living and mobility in elderly diabetic patients following hip fracture are significantly worsened compared to nondiabetics [12]. However, another study found that mobility in elderly hip fracture patients with diabetes is similar to that of nondiabetics at 1 year after injury, while diabetics are at an increased risk of cardiac and pressure ulcers as postoperative complications [13]. The reason for the discrepancy in these studies is not fully understood. However, several studies have indicated that different ages, sexes among groups, the ratio of diabetic patients in the sample, preinjury activity, medical system, and socioeconomic conditions may contribute at least partly to diverse outcomes after injury [4, 14–16]. For example, Martin-Martin et al. found that advanced age is more likely associated with poorer functional recovery following hip fracture [14]. Kristensen et al. reported that after hip fracture, men had a two-fold higher mortality than women [15]. In addition, differences in baseline functional status in older adults with diabetes were associated with outcome differences [16].

To further clarify the relation between diabetes and outcomes after fragility hip fracture, we conducted a retrospective cohort study involving 161 diabetic hip fracture patients and 483 nondiabetic hip fracture patients [17]. Follow-up assessments were done on day 30 and 1 year after fracture. The aim of our study was to explore the effect of diabetes on short-term (30 days after fracture) and 1-year outcomes for fragility hip fracture patients and to determine whether there were any changes that could be made in clinical practice to improve patient recovery in the Chinese population.

## 2. Methods

### 2.1. Study Population

We conducted a retrospective cohort study involving fragility hip fracture patients who attended the First Affiliated Hospital of Chongqing Medical University between June 2013 and July 2016. The patients were categorized into nondiabetic group and diabetic group and were followed up on day 30 and 1 year after fracture. A total of 644 patients were included in the analysis. We prescreened patients according to the following criteria: (1) age ≥60 years and (2) a fall from standing height or less. The following exclusion criteria were applied: (1) high-impact injury, such as traffic accidents, high fall accident, and multiple traumas and (2) patients with pathological fractures.

The patients were classified into two groups according to whether there was diabetes or not. Diabetes was considered to be present when the following criteria were fulfilled at admission: (1) the patient had a diagnosis of diabetes previously established by a diabetologist and (2) HbAlc ≥6.5% and fasting plasma glucose ≥7.0 mmol/L (fasting is defined as no caloric intake for at least eight hours), classic symptoms of hyperglycaemia or hyperglycaemic crisis, and a random plasma glucose ≥11.1 mmol/L.

Our study was approved by the ethics review board of the First Affiliated Hospital of Chongqing Medical University.

### 2.2. Baseline Data

We collected the following data from patients' medical records: (1) demographics, such as age, gender, smoking habits, and alcohol misuse; (2) information related to diabetes (i.e., duration of diabetes and treatment measures); (3) laboratory test results and bone mineral density (BMD), including haemoglobin, serum albumin, estimated glomerular filtration rate (eGFR), BMD at the neck of the femur and BMD at the spine; (4) comorbidities, such as hypertension, coronary artery disease, cardiac failure, cerebrovascular disease, kidney disease, respiratory disease, gastrointestinal diseases, cancer, and Charlson comorbidity index; (5) information related to the fracture, i.e., history of previous fracture, mobility of prefracture, type of fracture, time awaiting surgery, and type of surgery; and (6) antiosteoporosis treatment, i.e., patients were prescribed antiosteoporosis medication after hip fracture and patients continued with their treatment by the end of the first year.

### 2.3. Definitions

Estimated GFR was calculated according to the Chronic Kidney Disease-Epidemiology Collaboration (CKD-EPI) equations [18]. For baseline registration of prefracture comorbidity, we used the Charlson comorbidity index. The scores range from 0 to 30, with a high score suggesting high comorbidity [19]. Prefracture and postfracture functional outcomes were evaluated using activity levels [20], which were defined as follows: I, normal; II, essentially independent outdoors but needs help with some outside activities; III, independent indoors but always requiring help outdoors; IV, not independent indoors but able to transfer and walk independently; and V, confined to bed or chair and not ambulatory. When there was an occurrence of reduced activity level, we registered the corresponding scores. For example, when someone was from grade I to grade IV, we registered 3.

### 2.4. Outcomes

Endpoint consisted of two parts: (1) short-term outcomes, i.e., complications that occurred within 30 days after fracture (cardiac diseases, cerebrovascular accidents, pneumonia, deep vein thrombosis, urinary tract infections, wound infection, pulmonary embolism, transient delirium, and pressure ulcer), length of stay, total cost, and the course of rehabilitation after surgery (discharge destination and duration of rehabilitation unit); (2) 1-year outcomes, i.e., postfracture functional outcomes and reduced activity level and mortality rate at 1 year after fracture.

### 2.5. Statistical Analysis

Statistical analysis was performed using SPSS version 21.0 (SPSS Inc., Chicago, IL, USA). Categorical variables are represented as numbers and proportions, and Fisher's exact test was used to compare proportions. We checked the normality of data by visual inspection of Q-Q plots. The normally distributed continuous variables are expressed as mean (standard deviation), and the differences were tested with Student's *t*-test. Nonnormally distributed variables are expressed as median (interquartile range (IQR)). The differences were tested with the Mann–Whitney *U* test. Rank variables are represented as numbers. The Mann–Whitney *U* test is used to compare the differences of rank data. All calculated *p* values were 2-tailed, with values <0.05 considered statistically significant.

## 3. Results

### 3.1. Characteristics of the Study Population

A database of 644 individuals was collated including 483 nondiabetic patients and 161 diabetic patients. In the whole cohort, 430 (66.8%) were females and 214 (33.2%) males, with average patient age of 77.9 ± 8.4 years. In the diabetic group there were 44 (27.4%) men and 117 (72.7%) women and the mean age was 77.3 ± 7.6 years in the diabetic group. The average duration of diabetes was 10.6 ± 7.8 years, including 44.8% of insulin therapy, 32.7% of drug therapy, and 22.4% of untreated patients. The diabetic patients showed the following: less smoking, higher serum albumin, lower eGFR, more hypertension, more kidney disease, and longer waiting time for surgery than nondiabetic patients. There were no significant differences between the groups in age, gender, alcohol, haemoglobin, BMD, Charlson comorbidity index, comorbidity (except hypertension and kidney disease), previous fracture, preinjury activity, fracture type, therapeutic regimen, surgical treatment, and antiosteoporosis medication (Table 1).

### 3.2. Short-Term Outcomes

Postfracture complications that occurred within 30 days after fracture are shown in Table 2. Diabetic patients had significantly higher rates of deep vein thrombosis (4.3% vs. 1.4%, *P*=0.029) and urinary tract infections (6.2% vs. 1.7%, *P*=0.002) compared to the nondiabetic patients. Nevertheless, there were no significant differences between groups for other complications, such as cardiac diseases, cerebrovascular accidents, pneumonia, wound infection, transient delirium, pressure ulcer, and pulmonary embolism.

The mean postoperative hospital stay and total hospital stay were significantly longer in the diabetic group than in the nondiabetic group (*P*=0.05, *P* < 0.01). In addition, total cost per patient in the diabetic group was also higher than that in the nondiabetic group (*P*=0.011) (Table 2). When looking at the course of rehabilitation after hip fractures, no significant difference in the kind and duration of rehabilitation after hip fracture was found between the patients with or without diabetes.

### 3.3. 1-Year Outcomes

At 1 year after fracture, of 644 patients, 31 were lost to follow-up and 48 (7.5%) died. We evaluated the ambulatory status and functional activity of the 565 (87.7%) patients who were alive and thus could be followed up at 1 year. However, only 40.9% of the patients were ambulant unaided and 59.1% needed ambulatory aid. No statistically significant difference between the functional activity was found. Moreover, we found that 54.1% patients in the diabetic hip fracture group and 56.3% in the nondiabetic hip fracture group failed to return to their prefracture mobility at 1-year follow-up. However, compared with nondiabetic patients diabetic patients did not have exacerbated decline of functional activity (54.1% vs. 56.3%, *P*=0.521) and did not present with increased 1-year mortality (5.6% vs. 8.1%, *P*=0.299). Data are presented in Table 3.

## 4. Discussion

Hip fracture in elderly patients is a major health problem that occurs frequently in most countries, resulting in greater mortality, significant disability, reduced quality of life, and even increased 1-year mortality [1, 3, 4, 14]. Compelling evidence has indicated that functional outcomes after hip fracture are variable according to the difference of age, sex, preinjury activity, and comorbid diseases. Diabetes seen as one recognizable, treatable, and preventable comorbid disease in geriatric adults may affect the rehabilitation process of hip fracture. Although more and more studies have confirmed that diabetes, especially type 2 diabetes, significantly increases risk of hip fracture, the characteristics and functional outcomes of diabetic hip fracture patients remain a source of debate. In our study, we partly excluded confounding factors and found that diabetic patients showed the following: less smoking, higher serum albumin, lower eGFR, more hypertension, more kidney disease, longer waiting time for surgery, longer length of hospital stay, and higher costs compared with nondiabetic patients. Furthermore, at 30 days after fracture, diabetic patients had significantly higher rates of urinary tract infections and deep vein thrombosis than nondiabetic patients. However, no differences in functional outcome and 1-year mortality between the groups were found during the study period.

In our study, the number of female patients (66.8%) was significantly greater than that of male patients (33.2%). These results are in line with those of previous studies [21, 22]. Although diabetes was associated with significantly increased risk of hip fracture, a previous meta-analysis reported that the risk of hip fractures is more pronounced in men with diabetes [10]. On the other hand, this outcome is contrary to that of Hothersall's study [23] which found an increased risk of hip fractures among diabetic women only. Some meta-analysis reported that the association between diabetes and hip fracture risk was similar in both genders [24, 25]. In this study, 161 patients had diabetes mellitus with 117 (72.7%) in women and 44 (27.3%) in men. The reason for the discrepancy may be because of different socioeconomic conditions, geographical differences, or different medical systems.

A variety of complications are often observed in elder patients with hip fracture. However, only a small part of them are operation-correlated complications, such as wound infection and migration and breakage of implants. The most common complications are medical diseases, such as pneumonia, delirium, urinary tract infection, and heart failure. In our study, the most common complications that occurred within 30 days after fracture were pneumonia (4.5%), urinary tract infection (2.8%), and deep vein thrombosis (2.2%). The result was somewhat different from the findings of Roche et al. [26], who found that the three most encountered medical complications were chest infection (9%), heart failure (5%), and urinary tract infection (4%). However, another study reported that the most common nonsurgical complications were delirium (20%), followed by pneumonia (10%) and heart failure (5%) [27]. Complication rates after hip fracture reported in the literature vary considerably. Possible causes of these differences in rates are study design, time to follow-up, the operation time, etc. Furthermore, we found that diabetic hip fracture patients had significantly higher rates of deep vein thrombosis than patients in the nondiabetic hip fracture group. An explanation for the higher incidence could be the longer time awaiting surgery and more comorbidities in diabetic patients. Thus, we should balance the attractions of the probable benefits of postponement against the disadvantages of prolonged discomfort and the possible complications. Urinary tract infections are among the relatively common bacterial infections in elderly patients with hip fractures. Moreover, diabetes is a risk factor for urinary tract infections [28]. Similarly, hip fracture patients with diabetes had a higher rate of urinary tract infection complications in our study. Therefore, it is crucial to assess and identify urinary tract infection symptoms in patients with diabetes.

Mobility was selected as the functional outcome because older patients with hip fractures often have a marked and permanent deterioration in their walking ability [29]. In our study, we found that 54.1% patients in the diabetic hip fracture group and 56.3% in the nondiabetic hip fracture group failed to return to their prefracture mobility at 1-year follow-up. This result is similar to the results of earlier research [30], which found that 42% of patients are incapable of returning to their prefracture mobility and 35% are unable to walk independently during the first year after hip fracture. Surprisingly, in our study, diabetes had no influence on the change in mobility and mortality at 1 year after hip fracture. Identical results have also been declared in a study with a follow-up of 2 years after hip fracture [31]. However, Lieberman et al. demonstrated a poorer functional status following hip fracture in diabetic vs. nondiabetic patients [12]. The reason for the discrepancy may be because of different instruments of functional assessment or different follow-up times. In our study, considering the retrospective design and practicality in follow-up, we used Activity Level scale instead of Functional Independence Measure (FIM) scale. The FIM measures independent performance in self-care, sphincter control, transfers, locomotion, communication, and social cognition. It describes a person's functional abilities and limitations in activities required for daily living. Thus, proper instruments of functional assessment and long-term follow-up are warranted to better achieve the goal of clarifying the association between diabetes and functional outcomes after hip fracture in elderly patients.

There are some limitations in this study. First, the dataset used in this study was generated from one hospital, limiting its generalizability to other hospitals; therefore, additional large-scale research is needed. Second, due to the retrospective design of our study, we used concise and practical Activity Levels to evaluate functional outcomes. This is a possible determinant of the different outcomes found. Then, some other factors can influence functional outcomes after a fragility fracture, such as poor vitamin D status, BMI, obesity, and income level. Finally, because of the limited sample and time of follow-up, there may be false-negative results on the influence of diabetes on mobility.

## 5. Conclusion

Compared with nondiabetic patients diabetic patients have more comorbidity, and once hip fracture occurs, they have longer waiting time for surgery, higher costs, and significantly increased risks for developing urinary tract infections and deep venous thrombosis complications. However, diabetic patients do not have worsened functional outcomes and higher 1-year mortality compared to nondiabetic patients. Proper long-term follow-up may be needed to better clarify the association between diabetes and functional outcomes after hip fracture in elderly patients in order to take measures earlier to prevent and treat diabetes.

## Figures and Tables

**Table 1:** Comparison of general characteristics of the two groups of patients.

	Nondiabetic (*N* = 483)	Diabetic (*N* = 161)	*P* value
Demographics			
Age (years)	78.0 ± 8.6	77.3 ± 7.6	0.310
Gender (*n* (%))			0.066
Male	170 (35.2)	44 (27.4)	
Female	313 (64.8)	117 (72.7)	
Substance use (*n* (%))			
Smoking habit	81 (16.8)	13 (8.1)	0.007
Alcohol misuse	51 (10.6)	17 (10.6)	1.000
Laboratory test			
Haemoglobin (g/l)	113.0 ± 19.2	114.0 ± 20.4	0.568
Serum albumin (g/l)	36.8 ± 4.6	37.6 ± 4.0	0.035
eGFR (ml min^−1^·1.73 m^−2^)	75.4 ± 21.1	70.6 ± 24.3	0.027
BMD at the neck of the femur (g/cm^3^)	0.55 ± 0.22	0.59 ± 0.22	0.193
BMD at the spine (g/cm^3^)	0.50 ± 0.35	0.54 ± 0.37	0.255
Charlson comorbidity index (*n* (%))			0.891
0–2	441 (91.3)	145 (90.1)	
3-4	39 (8.1)	15 (9.3)	
5–6	3 (0.6)	1 (0.6)	
Mean	0.7 ± 1.0	0.9 ± 1.2	0.051
Comorbidity (*n* (%))			
Hypertension	195 (40.4)	114 (70.8)	<0.001
Coronary artery disease	69 (14.3)	37 (23.0)	0.010
Cardiac failure	29 (6.0)	10 (6.2)	0.924
Cerebrovascular accidents	68 (14.1)	29 (18.0)	0.227
Kidney disease	23 (4.8)	18 (11.2)	0.004
Respiratory disease	63 (13.0)	19 (11.8)	0.682
Gastrointestinal diseases	32 (6.6)	5 (3.1)	0.176
Cancer	27 (5.6)	9 (5.6)	1.000
Previous fracture (*n* (%))	55 (11.4)	18 (11.2)	0.943
Preinjury activity (*n* (%))			0.348
Grade I	380 (90.7)	134 (91.8)	
Grade II	31 (7.4)	9 (6.2)	
Grade III	8 (1.9)	2 (1.4)	
Grade IV	0 (0)	1 (0.7)	
Fracture type (*n* (%))			0.689
Femoral neck	282 (58.6)	92 (57.1)	
Trochanteric	193 (40.0)	65 (40.4)	
Subtrochanteric	7 (1.4)	4 (2.5)	
Time awaiting surgery (days)	5.0 (4.0–8.0)	6.0 (5.0–8.0)	0.031
Therapeutic regimen (*n* (%))			0.47
No surgery	106 (21.9)	31 (19.3)	
Underwent surgery	377 (78.1)	130 (80.7)	
Surgical treatment (*n* (%))			0.238
Internal fixation	76 (20.2)	34 (26.2)	
Hemiarthroplasty	153 (40.6)	54 (41.5)	
Total hip replacement	148 (39.3)	42 (32.3)	
Antiosteoporosis medication (*n* (%))			
Prescribed after hip fracture	146 (34.8)	50 (34.2)	0.896
Continuous users in the 12 months	80 (19.1)	25 (17.1)	0.598

Data are represented as mean (SD), median (IQR), or *n* (%). eGFR, estimated glomerular filtration rate; BMD, bone mineral density.

**Table 2:** Comparison of short-term outcome after fracture in the two groups of patients.

	Nondiabetic (*N* = 483)	Diabetic (*N* = 161)	*P* value
Complications			
Cardiac diseases (*n* (%))	9 (1.9)	2 (1.2)	0.586
Cerebrovascular accidents (*n* (%))	2 (0.4)	0 (0)	0.283
Pneumonia (*n* (%))	20 (4.1)	9 (5.6)	0.443
Deep vein thrombosis (*n* (%))	7 (1.4)	7 (4.3)	0.029
Urinary tract infections (*n* (%))	8 (1.7)	10 (6.2)	0.002
Wound infection (*n* (%))	3 (0.6)	1 (0.6)	1.000
Pulmonary embolism (*n* (%))	3 (0.6)	1 (0.6)	1.000
Transient delirium (*n* (%))	6 (1.2)	1 (0.6)	0.485
Pressure ulcer (*n* (%))	6 (1.2)	1 (0.6)	0.485
Postoperative hospital stays (days)	9.00 (7.0–12.0)	11.00 (7.0–15.0)	0.05
Total hospital stays (days)	15.0 (12.0–19.0)	17.0 (14.0–23.5)	<0.001
Total cost (CNY × 10^4^)	11.5 (6.2–14.0)	13.0 (8.4–15.7)	0.011
Discharge destination (*n* (%))			0.377
Rehabilitation unit	32 (6.6%)	14 (8.7%)	
Home	451 (93.4%)	147 (91.3%)	
Duration of rehabilitation unit duration	32 (21.3–51.5)	29 (24.0–53.0)	0.830

Data are represented as median (IQR) or *n* (%). CNY, Chinese yuan.

**Table 3:** Comparison of 1-year outcome and mortality after fracture in the two groups of patients.

	Nondiabetic	Diabetic	*P* value
Activity at final follow-up	419	146	0.302
Grade I	168 (40.1)	63 (43.2)	
Grade II	139 (33.2)	45 (30.8)	
Grade III	88 (21.0)	24 (16.4)	
Grade IV	12 (2.9)	9 (6.2)	
Grade V	12 (2.9)	5 (3.4)	
Change in mobility score (≥1)	236	79	0.521
1	141 (59.7)	46 (58.2)	
2	80 (33.9)	22 (27.8)	
3	9 (3.8)	10 (12.7)	
4	6 (2.5)	1 (1.3)	
Mortality at one-year follow-up	39 (8.1)	9 (5.6)	0.299

## Data Availability

All the data needed to achieve the conclusion are presented in this paper. The raw data used to support the findings of this study are available from the corresponding author upon request.

## Abbreviations

BMD: Bone mineral density
eGFR: Estimated glomerular filtration rate
CKD-EPI: Chronic Kidney Disease-Epidemiology Collaboration
IQR: Interquartile range
CNY: Chinese yuan
FIM: Functional independence measure
BMI: Body mass index.
